# Geographical Origin Identification of *Dendrobium officinale* Using Variational Inference-Enhanced Deep Learning

**DOI:** 10.3390/foods14193361

**Published:** 2025-09-28

**Authors:** Changqing Liu, Fan Cao, Yifeng Diao, Yan He, Shuting Cai

**Affiliations:** 1 School of Automation, Guangdong University of Technology, Guangzhou 510006, China; 1112304030@mail2.gdut.edu.cn (C.L.); 1112204013@mail2.gdut.edu.cn (F.C.); 2 School of Integrated Circuits, Guangdong University of Technology, Guangzhou 510006, China; 2112304102@mail2.gdut.edu.cn; 3 School of Biomedical and Pharmaceutical Sciences, Guangdong University of Technology, Guangzhou 510006, China; heyan129@gdut.edu.cn

**Keywords:** *Dendrobium officinale*, geographical origin identification, image classification, machine learning, variational inference, non-destructive testing

## Abstract

*Dendrobium officinale* is an important medicinal and edible plant in China, widely used in the dietary health industry and pharmaceutical field. Due to the different geographical origins and cultivation methods, the nutritional value, medicinal quality, and price of *Dendrobium* are significantly different, and accurate identification of the origin is crucial. Current origin identification relies on expert judgment or requires costly instruments, lacking an efficient solution. This study proposes a Variational Inference-enabled Data-Efficient Learning (VIDE) model for high-precision, non-destructive origin identification using a small number of image samples. VIDE integrates dual probabilistic networks: a prior network generating latent feature prototypes and a posterior network employing variational inference to model feature distributions via mean and variance estimators. This synergistic design enhances intra-class feature diversity while maximizing inter-class separability, achieving robust classification with limited samples. Experiments on a self-built dataset of *Dendrobium officinale* samples from six major Chinese regions show the VIDE model achieves 91.51% precision, 92.63% recall, and 92.07% F1-score, outperforming state-of-the-art models. The study offers a practical solution for geographical origin identification and advances intelligent quality assessment in *Dendrobium officinale*.

## 1. Introduction

*Dendrobium officinale*, a perennial herb of the Orchidaceae family, predominantly inhabits warm, humid, semi-shaded environments at elevations of 100–3000 m [1]. This economically valuable species serves dual medicinal and dietary purposes, demonstrating pharmacological activities encompassing gastroprotection, hypotensive effects, antitumor properties, and neural regeneration [2,3,4]. Driven by substantial market demand, the industrial cultivation of *Dendrobium officinale* in China has expanded considerably. It was reported that China’s annual production of fresh stems reaches approximately 60,000 tonnes, supporting a substantial domestic market worth over USD 4 billion (https://www.chinabgao.com/info/1275021.html, accessed on 17 September 2025). Modern pharmaceutical standards necessitate comprehensive quality control protocols spanning cultivation traceability, bioactive compound analysis, and pathogen management. The plant’s active ingredient profiles exhibit marked variations across geographic origins due to climatic conditions and cultivation practices, directly influencing yield metrics, market valuation, and therapeutic efficacy [5,6,7]. These origin-dependent quality disparities, coupled with significant price differentials, underscore the imperative for precise geographical authentication in pharmacological applications.

Traditional methods for identifying the geographical origins of medicinal and edible plants, especially Chinese herbal medicines, primarily depend on expert morphological evaluations, involving multidimensional assessment of characteristics including shape, length, width, surface texture, color, and organoleptic properties such as odor and taste [8]. This approach demonstrates inherent subjectivity, requiring experts to accumulate years of practical experience to discern origin-specific variations. External environmental factors including lighting conditions, temperature fluctuations, and humidity levels further compromise reproducibility. The reliance on qualitative descriptors (e.g., “bitter–slightly sweet taste”, “fragrant smell”) precludes the establishment of a quantifiable and standardized evaluation system.

Advancements in analytical chemistry have facilitated multimodal identification technologies: molecular-level analyses (DNA barcoding, NMR-based metabolite profiling), chemical composition characterization (HPLC quantification of signature compounds, GC-MS analysis of volatile components), and spectral feature modeling (NIR spectroscopy with chemometrics, ATR-FTIR fingerprinting) [9]. While these techniques enhance identification precision, their implementation faces limitations including technical complexity requiring specialized operators, limited accessibility in rural testing facilities, prohibitive instrumentation costs, inter-instrument compatibility issues, and sample destructiveness during analysis [10,11].

Artificial intelligence technology provides a key solution to the technical bottlenecks in medicinal and edible plant analysis, particularly regarding operator expertise dependency, analytical costs, data standardization challenges, and sample destructiveness [12]. This convergence enables intelligent modeling for precise analysis of complex botanical systems through cost-effective computational frameworks. Cai et al. [13] developed a hyperspectral–deep learning hybrid system for chrysanthemum classification, employing convolutional neural networks with few-shot incremental learning to mitigate sample scarcity (30 incremental samples) and class imbalance (4→14 classes), maintaining 80.13% accuracy. Lu et al. [14] established a Raman spectroscopy authentication protocol for Astragalus membranaceus combining PCA and PLS-DA, demonstrating efficacy in geographical authentication of heterogeneous herbal materials. Ding et al. [15] innovated group intelligence decision-making systems for cultivation optimization, while Miao et al. [16] enhanced the ConvNeXt architecture with ACMix and FFN networks to improve the feature extraction of herbal medicines. Complementing these, Gao et al. [17] implemented a computer vision-driven SVM model using colour features, texture features and shape features of Chinese herbal medicine slice images for quality grading.

Near-infrared spectroscopy and Raman spectroscopy coupled with artificial intelligence have demonstrated growing applications in *Dendrobium officinale* research. Gong et al. [18] developed an NMR–artificial neural network (ANN)-integrated approach for rapid adulterant identification and quantification in *Dendrobium officinale* powder. Li et al. [19] implemented ATR-FTIR spectroscopy with PLS-DA and SVM modeling, incorporating Savitzky–Golay preprocessing to evaluate organ-specific and temporal variations in bioactive compound accumulation. Their chemometric strategy established optimal stem harvesting periods (November–January) while resolving botanical part differentiation challenges. She et al. [20] established an NIR-based quality control framework utilizing total saponins, mannitol, and naringenin as phytochemical markers. Spectral normalization coupled with UVE-CARS wavelength optimization enhanced model robustness. Zhang et al. [21] analyzed 200 *Dendrobium officinale* samples via Raman spectroscopy, comparing single classifiers (KNN, MLP, DTC) with stacked ensembles. The neural network with random weight (NNRW) ensemble achieved peak performance (96.3% accuracy) through gradient-free computation and bias reduction, establishing a novel spectral authentication paradigm.

Although machine learning enhances spectral analysis through automated feature extraction and cross-platform data calibration, spectral methodologies still possess inherent limitations. These limitations include susceptibility of spectral signals to environmental noise, insufficient resolution due to overlapping characteristic bands, and the need for complex sample preprocessing—all of which restrict rapid on-site testing applications. In contrast to spectral analysis, machine learning-based plant phenotyping approaches can better integrate multi-source heterogeneous data, extract deep phenotypic features, and establish nonlinear predictive models, thereby improving classification accuracy and robustness. Notably, in the geographical origin authentication of *Dendrobium officinale*, variations in geographical composition exhibit weaker discriminative power compared to interspecies differences, and this process is further challenged by sample acquisition costs, such as insufficient sample size and minimal regional variation. To address these challenges, we present VIDE (Variational Inference-enabled Data-Efficient Learning), a novel framework designed to mitigate data scarcity and enhance model generalization in machine learning. The proposed approach establishes a fast, cost-effective, and non-destructive solution for origin traceability in *Dendrobium officinale*. The main contributions of this study are as follows:

(1) This study addresses the challenges of minimal inter-class feature disparity and restricted intra-class sample availability in *Dendrobium officinale* image classification by implementing a variational inference-driven latent feature enhancement strategy. The dual-network architecture synergizes prior botanical knowledge extraction with posterior feature generation under Gaussian distribution constraints, enforcing compact intra-class feature clustering and enhanced inter-class separability for improved discriminative learning.
(2) To mitigate overfitting, the framework integrates residual-based convolutional blocks with reparameterization techniques, achieving lightweight deployment and end-to-end optimization. Low-cost, high-efficiency origin identification of samples can be accomplished simply by analyzing images captured by smartphones, breaking through the limitations of traditional identification methods that rely on high-cost equipment (e.g., hyperspectral technology) or expert experience.
(3) Validation through a multi-source image database demonstrates statistically significant improvements in geographical origin classification accuracy compared to conventional models, confirming the framework’s efficacy and generalizability.

The rest of this paper is organized as follows. Section 2 details the new method we proposed. Section 3 presents the experimental results and analysis. Section 4 discusses the proposed method. Finally, Section 5 concludes this study.

## 2. Materials and Methods

### 2.1. Image Acquisition

**Figure 1:**
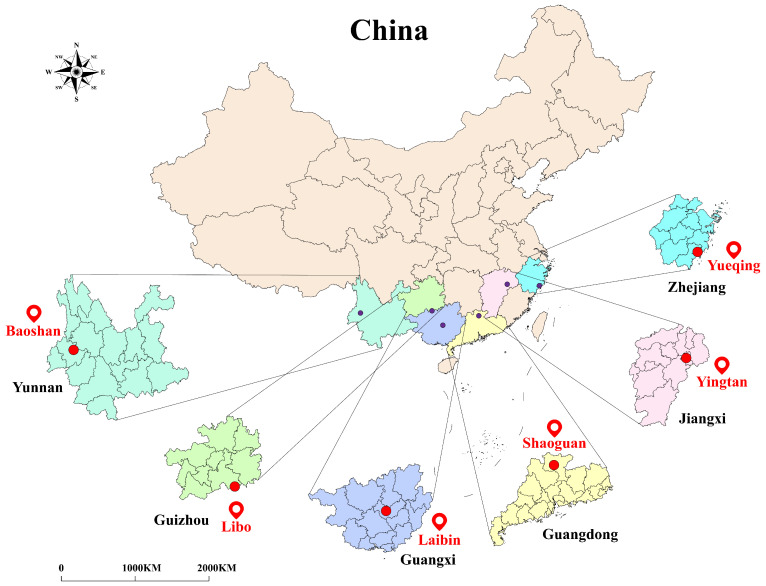
Geographical origin of the *Dendrobium officinale* samples.

**Figure 2:**
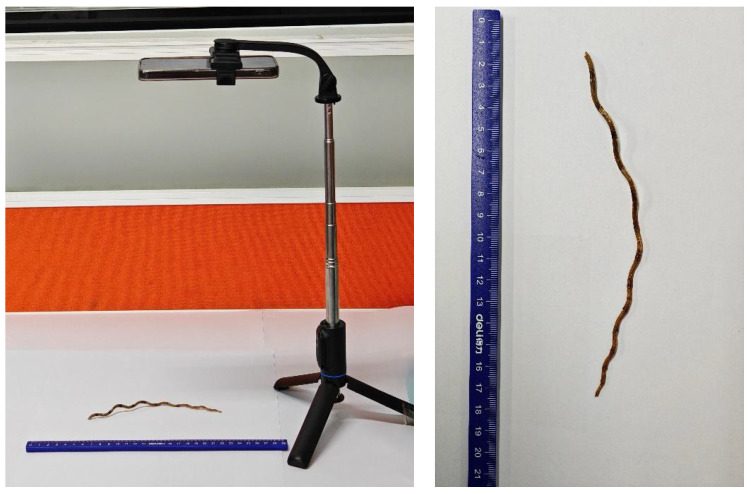
Imaging platform for *Dendrobium officinale* sample acquisition.

**Figure 3:**
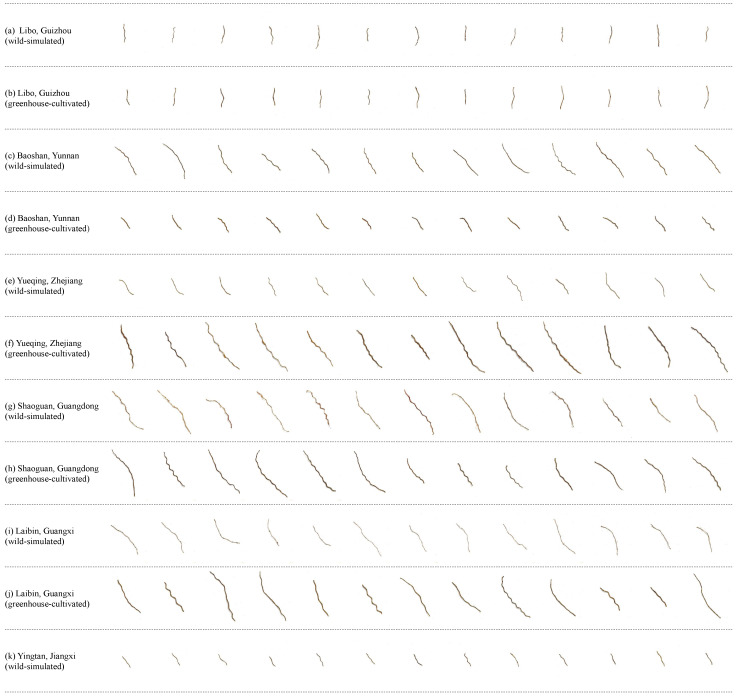
Dataset examples of *Dendrobium officinale* from different production regions.

The stem samples of *Dendrobium officinale* used in this study were collected from multiple regions across China, including Libo (Guizhou), Baoshan (Yunnan), Yueqing (Zhejiang), Shaoguan (Guangdong), Laibin (Guangxi), and Yingtan (Jiangxi). The geographical distribution of these samples is shown in Figure 1. The samples were collected in January 2024 and included 11 batches, with each batch containing 200 individual samples. The cultivation methods included two modes: wild-simulated planting and greenhouse cultivation. Cultivation practices, though varying by region, were standardized within each production area and aligned with local agricultural standards. This variation reflects real-world growing conditions and was treated as an integral aspect of geographical identity in this study. All the samples were authenticated as the stems of *Dendrobium officinale*. After collection, the samples were dried at low temperature, then placed in airtight bags and stored in a cool place. Detailed information of the samples is shown in Table 1 and Table 2. Image acquisition was conducted indoors under natural light conditions. As shown in Figure 2, each *Dendrobium officinale* sample was placed on a white background plate to ensure a clear contrast between the foreground target and the background for subsequent identification. The experiment was performed using a realme GT5 Pro smartphone (equipped with a Sony LYT-808 rear main camera, 50MP maximum pixel resolution). The smartphone camera was fixed 45 cm above the samples and kept horizontal. Images were collected from September to October, 2024, from 9:00 a.m. to 11:00 a.m. at the laboratory of Guangdong University of Technology. The indoor environment was dry and relied only on natural light without using additional light sources. The camera was configured with a 3× zoom factor while maintaining consistent positioning for all samples. The camera was set to a magnification factor of 3× and ensured that all samples were taken in the same position. For each batch of samples, the images were captured separately with a resolution of 6144×8192 pixels and was saved in JPG format with clear details. Examples of each origin image dataset are shown in Figure 3.

The dataset constructed by the above method provides high-quality image resources of *Dendrobium officinale* for subsequent studies and ensures the reliability and consistency of the experimental data.

### 2.2. Symbol Definitions

The *Dendrobium officinale* images from the same sample space are partitioned into training dataset and test dataset. Let $D={\left\{\left(x_{i},y_{i}\right)\right\}}_{i=1}^{n}$ denote the training dataset, where *n* represents the number of samples in the training dataset. For any sample, $x\in R^{W\times H\times C}$ within D corresponds to an acquired image, where *W* denotes the horizontal pixels of the image, *H* denotes the vertical pixels of the image, and *C* denotes the number of channels of the image. The $y_{i}$ is the corresponding sample category label. Assuming that a certain sample $x_{i}$ has a total of *L* categories, we perform one-hot coding on the discrete categorical labels, and convert them into an *L*-dimensional vector $y\in {\left\{0,1\right\}}^{L}$ in binary representation. Similarly, we can represent the test dataset as $D^{t}={\left\{(x_{i},y_{i})\right\}}_{i=1}^{m}$, where *m* denotes the number of samples in the test dataset. In the test task, a judgment needs to be made about the category to which each sample belongs. When the input sample belongs to the *l*-th class(l∈L), the *l*-th element in the label vector *y* is 1, and the other elements values are 0. For convenience, we let (x,y) denote any sample and its corresponding category label.

### 2.3. VIDE Model Framework

**Figure 4:**
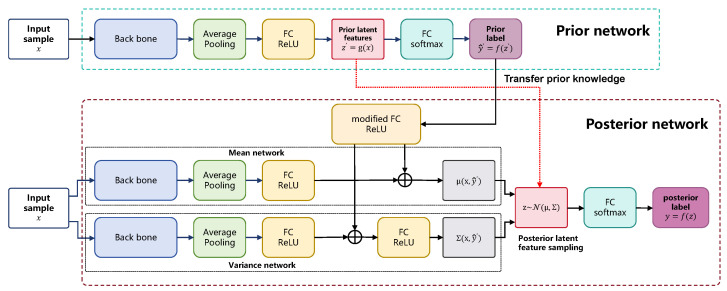
The complete network architecture of our proposed VIDE.

In this study, we propose a Variational Inference-enabled Data-Efficient Learning framework (VIDE) for geographical origin identification of *Dendrobium officinale*. The framework of our proposed VIDE model is shown in Figure 4. The VIDE model is mainly composed of two parts: a prior network and a posterior network.

#### 2.3.1. Prior Network

In the prior network, the high-dimensional *Dendrobium officinale* images *x* are acquired as the input images and generate the prior latent features by a feature extractor. Let g(·) denote the mapping function from the input images to latent variables, then the generated prior latent features can be represented as $z^{\prime }=g\left(x\right)\in R^{d_{z}}$, where $d_{z}$ represents the dimension of the prior latent features. Further, these prior latent features $z^{\prime }$ are then passed to subsequent network layers, and generate the corresponding prior labels ${\hat{y}}^{\prime }=f\left(z^{\prime }\right)={\left[{\hat{y}}_{1}^{\prime },{\hat{y}}_{2}^{\prime },\ldots ,{\hat{y}}_{L}^{\prime }\right]}^{T}$ by the classifier f(·).

#### 2.3.2. Posterior Network

Inspired by the variational autoencoder (VAE) [22], we construct the posterior network consisting of a pair of twinned convolutional neural networks μ(·) and σ(·), where μ(·) is the mean network, and σ(·) is the variance network. In the posterior network, the input image *x* and its corresponding prior label ${\hat{y}}^{\prime }$ are fed into these two independent neural networks, respectively, obtaining two vectors: μ(x) and σ(x). We assume that the conditional probability distribution $Q(z|x,{\hat{y}}^{\prime })$ of the posterior latent features $z\in R^{d_{z}}$ follows a Gaussian distribution, whose probability density function can be expressed as

(1) $$\begin{array}{c} Q\left(z|x,{\hat{y}}^{\prime }\right)=N\left(\mu \left(x,{\hat{y}}^{\prime }\right),\Sigma \left(x,{\hat{y}}^{\prime }\right)\right) \end{array}$$

where $\mu \left(x,{\hat{y}}^{\prime }\right)\in R^{d_{z}}$ is the mean of the Gaussian distribution, $\Sigma \left(x,{\hat{y}}^{\prime }\right)\in R^{d_{z}\times d_{z}}$ represents the covariance matrix, and $d_{z}$ is the dimension of posterior latent features. Through sampling from the latent feature distribution $N\left(\mu \left(x\right),\Sigma \left(x\right)\right)$, a large number of posterior latent features *z* can be obtained, which can be specifically expressed as

(2) $$\begin{array}{c} z∼N\left(\mu \left(x,{\hat{y}}^{\prime }\right),\Sigma \left(x,{\hat{y}}^{\prime }\right)\right) \end{array}$$

where the covariance matrix $\Sigma \left(x,{\hat{y}}^{\prime }\right)=\mathrm{diag}\left(\sigma (x,{\hat{y}}^{\prime })\right)$ is a diagonal matrix, and the elements on the diagonal are $\sigma \left(x,{\hat{y}}^{\prime }\right)\in R^{d_{z}}$. The posterior latent features *z* obtained from these samples are then input to the posterior-learning-based decoder $P\left(y|\cdot \right)$ for decoding, which in turn yields the predicted value of the posterior relationship $\hat{y}$ for the input samples.

(3) $$\begin{array}{c} \hat{y}=P\left(y|z\right) \end{array}$$

Unlike traditional convolutional neural networks, we use the mean and variance networks to solve the parameters of the latent variable distribution, then construct the latent feature distribution and obtain a large number of a posterior latent features from the sampling, which realizes the purpose of latent feature enhancement. The variance network has the same structure as the mean network, and the forward propagation algorithm for the VIDE model is described in Algorithm 1.
**Algorithm 1** Forward Propagation of VIDE Model**Require:**
Training set D, Test set $D^{t}$
**Ensure:**
$\hat{y}$
  1:Input samples to the prior network to obtain prior latent features $z^{\prime }$ and predicted labels ${\hat{y}}^{\prime }$  2:Feed samples with attached prior labels $\left(x,{\hat{y}}^{\prime }\right)$ into the posterior network to get $Q(z|x,{\hat{y}}^{\prime })$  3:**if** in testing phase **then**  4:    **for** each $\left(x_{i},y_{i}\right)\in D^{t}$ **do**  5:        Calculate $z=\mu (x,{\hat{y}}^{\prime })$  6:        Decode *z* using decoder P(y|·) to output posterior class label $\hat{y}=P\left(y|z\right)$  7:    **end for**  8:**end if**  9:**if** in training phase **then**10:    **for** t=1toT **do**11:        Obtain $\mu (x,{\hat{y}}^{\prime })$ and $\Sigma (x,{\hat{y}}^{\prime })$ from mean and variance networks12:        Sample posterior-augmented latent feature *z* from $N(\mu \left(x,{\hat{y}}^{\prime }\right),\Sigma \left(x,{\hat{y}}^{\prime }\right))$ according to Equation (1)13:        Calculate $\hat{y}=P\left(y|z\right)$14:    **end for**15:**end if**


### 2.4. Backbone of the VIDE Model

The backbone of the VIDE model is built upon a ResNet-based convolutional neural network architecture, which we have adapted to better suit the specific requirements of *Dendrobium officinale* image analysis. An RGB image of size H×W×3 is initially processed through a 3×3 convolutional layer followed by a 1×1 convolutional layer that acts as a fully connected layer, helping to reduce the parameter size of the optimized model and alleviate overfitting during training. Max-pooling layers are incorporated to improve feature messaging. The network is deepened using four convolutional modules, each containing two residual blocks with shortcut connections. Every residual block comprises two convolutional layers with the same number of output channels, each followed by batch normalization and a ReLU activation function to strengthen gradient flow during backpropagation. The shortcut connection bypasses these two convolutional operations and adds the input directly before the final ReLU activation. A 1×1 convolutional layer within the shortcut connection is employed to adjust dimensions flexibly, ensuring alignment with the output of the main pathway and enabling effective residual learning. This design not only mitigates gradient vanishing and computational overhead due to depth, but also promotes cross-channel interaction and fusion, thereby strengthening the network’s capacity to learn complex features while preserving stability and performance. The residual block is formalized as

(4) $$x_{i}^{\prime }=\phi \left(x_{i}\right)+h\left(x_{i}\right),$$

where $x_{i}$ and $x_{i}^{\prime }$ denote the input and output vectors of the residual block, ϕ(·) represents the residual mapping to be learned, and $h(x_{i})$ corresponds to the output of the 1×1 convolutional transformation applied to $x_{i}$.

### 2.5. Variational Inference Optimization

In the true posterior latent feature distribution, each posterior latent feature *z* can effectively represent the corresponding sample in the training dataset and each posterior latent feature sampled from this distribution can generate the corresponding original image and its associated class label. This posterior latent feature distribution is obtained from the samples in the training set by an encoder. Specifically, the obtained posterior latent feature distribution obeys the conditional probability P(z∣x,y). However, given a fixed training set, the above conditional probability distribution P(z∣x,y) is not observable. To estimate the parameters of the decoder described above, we use an approximate probability distribution Q(z) to fit the true posterior distribution P(z∣x,y). We use the Kullback–Leibler divergence (KL-divergence) [23] to describe the distance between the probability distribution Q(z) and the conditional probability distribution P(z∣x,y); thus, the difference between two probability density functions can be expressed as

(5) $$\begin{array}{c} D\left[Q\left(z\right)\|P\left(z|x,y\right)\right]=E_{z∼Q}\left[\log Q\left(z\right)-\log P\left(z|x,y\right)\right] \end{array}$$

Through Bayesian transformation, Equation (5) can be further expressed as

(6) $$\begin{array}{cc} \; & D\left[Q(z)\|P(z|x,y)\right]\\ \; & =E_{z∼Q}\left[\log Q(z)-\log P(z|x,y)\right]\\ \; & =E_{z∼Q}\left[\log Q\left(z\right)-\log P\left(x,y|z\right)-\log P\left(z\right)+\log P\left(x,y\right)\right]\\ \; & =E_{z∼Q}\left[\log Q\left(z\right)-\log P\left(x,y|z\right)-\log P\left(z\right)\right]+\log P\left(x,y\right) \end{array}$$

We aim to minimize the KL-divergence between Q(z) and P(z∣x,y) to ensure that the approximate distribution of the posterior latent features fits its true distribution. Subsequently, based on Equation (6), by merging and rearranging terms, we obtain the following equation:

(7) $$\begin{array}{c} \log P\left(x,y\right)-D\left[Q\left(z\right)∥P\left(z|x,y\right)\right]=E_{z∼Q}\left[\log P\left(x,y|z\right)+\log P\left(z\right)-\log Q\left(z\right)\right] \end{array}$$

Therefore, from Equation (7), we can derive the variational lower bound as

(8) $$\begin{array}{c} \log P(x,y)-D[Q(z)∥P(z|x,y)]\\ =E_{z∼Q}\left[\log P\left(x,y|z\right)\right]-E_{z∼Q}\left[\log Q\left(z\right)-\log P\left(z\right)\right]\\ =E_{z∼Q}\left[\log P\left(x,y|z\right)\right]-D\left[Q\left(z\right)∥P\left(z\right)\right] \end{array}$$

For computational convenience, the Gaussian distribution is a general assumption in variational inference methods [24]. Consequently, we assume the true distribution of the posterior latent features P(z) to be a regularized Gaussian distribution, expressed as

(9) $$\begin{array}{c} P\left(z\right)=N\left(\alpha {\tilde{z}}^{\prime },\beta I\right) \end{array}$$

where $I\in R^{d_{z}\times d_{z}}$ is a unit matrix; α and β are deflation factors used to adjust the mean and variance of the Gaussian distribution, respectively.

From Equation (9), it can be seen that the prior latent feature $z^{\prime }$ has a moderating effect on the true distribution P(z). This mechanism enables the posterior relational network to utilize the prior information provided by the prior relational network to generate posterior latent feature representations with enhanced robustness and distributional regularization. Here, ${\tilde{z}}^{\prime }\in R^{d_{z}}$ is the arithmetic mean of all prior latent features $z^{\prime }$ in the set S. The specific formula is as follows:

(10) $$\begin{array}{c} {\tilde{z}}^{\prime }=\frac{1}{\left|S\right|}\sum_{(x,y)\in S}g\left(x\right) \end{array}$$

where $\left|S\right|$ denotes the number of the small batch of samples acquired in each iteration. By averaging the latent prior features of the current batch samples, the impact of individual sample noise on the model is reduced, thereby enhancing generalization.

In the posterior network of the VIDE model, the approximate distribution Q(z) acts as an encoder that maps the observed data, i.e., the input data, into the space of the posterior latent feature distribution. Guided by the principle of amortized inference, we approximate the true posterior P(z∣x,y) using a variational distribution conditioned on the inputs. Specifically, the approximate distribution Q(z) in Equation (8) is replaced with a conditional probability $Q(z|x,{\hat{y}}^{\prime })$, leading to the following reformulated variational lower bound:

(11) $$\begin{array}{c} \log P\left(x,y\right)-D\left[Q\left(z|x,{\hat{y}}^{\prime }\right)∥P\left(z|x,y\right)\right]=E_{z∼Q}\left[\log P(x,y|z)\right]-D\left[Q\left(z|x,{\hat{y}}^{\prime }\right)∥P\left(z\right)\right] \end{array}$$

### 2.6. Objective Function

Based on the variational lower bound derived from variational inference, we can obtain the specific objective function of the VIDE model. Unlike traditional VAEs, our method is not concerned with the reconstruction loss of images or hidden features, but rather with whether the posterior-enhanced latent feature variables can be correctly identified by the decoder. To this end, we ignore the image reconstruction-related part of the decoder *P*, so the likelihood function $P\left(x,y|z\right)$ can be rewritten as $P\left(y|z\right)$. Consequently, the loss function is defined as

(12) $$\begin{array}{c} L\left(x,y;\omega \right)=-E_{\left(z,y)∼Q(z\right)}\left[\log P\left(y|z\right)]+D[Q\left(z|x,{\hat{y}}^{\prime }\right)∥P\left(z\right)\right] \end{array}$$

where ω represents the model parameters.

The first term is the expectation of the likelihood function under the approximate distribution of the posterior latent features, which we use as the cross-entropy loss function for the classification of *Dendrobium officinale* images. By minimizing the classification loss function, the VIDE model ensures that the prediction value of the posterior potential features is made to be the same as the given labels as much as possible, which in turn allows us to obtain a classifier with prediction ability, and ultimately to classify the input images. The logP is influenced by the posterior latent feature *z*. For the posterior latent feature *z*, it is sampled from the approximate distribution Q(z|x). This introduces stochastic units within the network, causing the backpropagation pathway of the model to be disconnected. To facilitate effective gradient backpropagation through the model, we employ the reparameterization trick [25], expressing the approximate distribution Q(z|x) as a function based on the standard Gaussian distribution. The posterior latent feature *z* can then be expressed as

(13) $$\begin{array}{cc} \; & z=\mu \left(x,{\hat{y}}^{\prime }\right)+\Sigma^{1/2}\left(x,{\hat{y}}^{\prime }\right)\cdot \varepsilon ,\\ \; & \varepsilon ∼N\left(0,I\right)\in R^{d_{z}} \end{array}$$

From the above equation, any Gaussian distribution can be represented as a function of a standard Gaussian distribution. After mapping the function, the random variables of the standard Gaussian distribution can be transformed into those of an arbitrary Gaussian distribution.

The second term is a regularization term for the latent variable distribution. By minimizing this term, the model ensures that the generated latent feature distribution $Q(z|x,{\hat{y}}^{\prime })$ approximate the true posterior latent feature distribution $N(\alpha {\tilde{z}}^{\prime },\beta I)$ as closely as possible. The position and bandwidth of the latent variable distribution are adjusted by scaling factors α and β. Since the KL-divergence between two Gaussian distributions has an analytical solution, we optimize the second term as follows:

(14) $$\begin{array}{c} D[Q\left(z|x,{\hat{y}}^{\prime }\right)∥P\left(z\right)]\\ \;=D[N\left(z;\mu \left(x,{\hat{y}}^{\prime }\right),\Sigma \left(x,{\hat{y}}^{\prime }\right)\right)∥N\left(\alpha {\tilde{z}}^{\prime },\beta I\right)]\\ \;=\frac{1}{2}\left[\mathrm{tr}\left({\left(\beta I\right)}^{-1}\Sigma \left(x,{\hat{y}}^{\prime }\right)\right)+\log\frac{det(\beta I)}{det(\Sigma \left(x,{\hat{y}}^{\prime }\right))}+{\left(\alpha {\tilde{z}}^{\prime }-\mu \left(x,{\hat{y}}^{\prime }\right)\right)}^{⊤}{\left(\beta I\right)}^{-1}\left(\alpha {\tilde{z}}^{\prime }-\mu \left(x,{\hat{y}}^{\prime }\right)\right)-d\right] \end{array}$$

In summary, in the task of classifying the geographical origin of *Dendrobium officinale*, for any of the category samples from set S, we minimize D[Q(z)∥P(z∣x,y)] by minimizing Equation (8). Then, the final loss function of the model can be obtained, as shown in Equation (12). Based on the two terms in Equation (12), we try to sample a large number of posterior latent features from the conditional probability distribution $Q(z|x,{\hat{y}}^{\prime })$, rather than computing explicit probability distribution expressions. This approach improves image classification accuracy under the condition of data-efficient learning.

## 3. Experimental Results

We conducted experiments on the collected data to investigate the recognition performance of our proposed method. In this study, all experiments were deployed on a graphics workstation equipped with a 2.90 GHz Intel Core i7 CPU, 64 GB RAM memory and NVIDIA GeForce RTX 4090 GPU with CUDA (version 11.8, Santa Clara, CA, USA). The operating system was Windows 11 and the algorithms were implemented using python 3.10 along with libraries such as pytorch (version 2.3.0), torchvision (version 0.18.0), and NumPy (version 1.18).

### 3.1. Experimental Details

The experiment involved comprehensive preprocessing of the acquired *Dendrobium officinale* image data. Specific operations included data normalization, denoising, random cropping, flipping, rotation, and scaling. Normalization ensured that the input samples conform to a similar distribution, thereby facilitating algorithm convergence. Denoising was applied to improve image quality. Random cropping, flipping, and rotation introduced perturbations to the input samples while achieving data augmentation, which enhanced the model’s robustness to noise and reduced its generalization error. Finally, all images were resized to a standardized scale to improve training efficiency. After completing these preprocessing steps, the dataset contained over 4000 images. These were divided into training (80%), validation (10%), and test (10%) sets using stratified sampling to preserve class distribution.

We divided the image samples into 11 categories according to the cultivation origin and cultivation method of *Dendrobium officinale*, i.e., 5 greenhouse cultivation from 5 different origins and 6 wild-simulated cultivation from different origins. To ensure the reliability of the results and to take into account the variability of the models, all the results in the experiments were the average of 10 experiments.

Specifically, the model framework was trained using the Adam optimizer to update the model parameters. The maximum training step size was set to 800, the initial learning rate was 0.001, and the learning rate was adjusted downward every 1/5 of the training step, with a learning rate decay factor of 0.1. In the experiments, the settings of the hyperparameter α were searched in the set $\left\{1.00,1.25,1.50,1.75,2.00,2.25,2.50,2.75,3.00,3.25,3.50\right\}$ and the settings of the hyperparameter β were searched in the set {0.01,0.25,0.50,0.75,1.00,1.25,1.50,1.75,2.00}. The parameterization of the baseline method was approximately the same as the original paper’s work.

### 3.2. Performance Comparison

We compared the performance of the proposed model with state-of-the-art models in the field of image classification to validate its advancement and feasibility. The baseline models we compared include Alexnet [26], VGGNet [27], GoogleNet [28], DenseNet121 [29], ResNet18 [30], ResNet50, ConvNeXt [31], EfficientNetV2 [32], and Vision Transformer (ViT) [33]. Each model was trained and tested using the same dataset. For the *Dendrobium officinale* dataset constructed in this study, we divided it into three subsets based on cultivation methods, a greenhouse cultivation dataset (samples from 5 origins), a wild-simulated cultivation dataset (samples from 6 origins), and a mixed cultivation dataset with greenhouse and wild-simulated samples. These subsets were used to test the performance of the models for the Geographical origin identification of *Dendrobium officinale*.

We used precision, recall, F1-score, and accuracy as evaluation metrics to measure the performance of the model and the results were shown in Table 3, Table 4 and Table 5. According to the performance and test results, the proposed model had the best performance compared to other baseline models on both the greenhouse cultivation dataset and the wild-simulated cultivation dataset. It reached precision, recall, F1-score, and accuracy of 95.41%, 95.83%, 95.62%, and 96.53%, respectively, for the greenhouse cultivation dataset and 94.37%, 93.92%, 94.14%, and 95.21% for the wild-simulated cultivation dataset. In particular, for the complex multi-source dataset containing greenhouse and wild-simulated cultivation methods, the model needed to trace the geographical origin of the *Dendrobium officinale* samples under different cultivation methods. In this task, the VIDE model still performed excellently, and its precision, recall, F1-score, and accuracy reached 91.51%, 92.63%, 92.07%, and 92.93%, respectively, which was significantly better than other advanced state-of-the-art image classification models. This is because the proposed model first extracts the feature representation of the samples from the prior network to obtain the prior latent variables. Then, the posterior network is used to map the input images with prior knowledge to the latent feature space and obtain the intra-class posterior latent feature probability distribution for each category of samples. By sampling from this distribution, we obtain the intra-class posterior latent features, capturing richer discriminative features and structural information in the intra-class samples while enhancing the inter-class variability among the various types of samples. Finally, the prediction results are decoded.

Based on the performance of other baseline models, we can further determine that AlexNet, VGGNet, and GoogleNet performed relatively weakly (72.65%, 76.23%, 79.06%), indicating that the traditional shallow convolutional structure has limited ability to capture the fine-grained features of *Dendrobium officinale*. In contrast, the residual networks ResNet18 and ResNet50, along with the densely connected architecture DenseNet121, improved the F1-score to 80.39%, 83.71%, and 82.22%, respectively, validating the effectiveness of feature reuse mechanisms. Among the advanced architectures, ConvNeXt (86.78%) and ViT (85.51%) significantly improve the performance by improving the convolutional operators or introducing the attention mechanisms, but there is still a score gap of about 6% compared to our proposed model. Specifically, ViT relies on large-scale data to capture long-range dependencies, while self-attention mechanisms become ineffective under small-data regimes. ConvNeXt borrows from ViT designs (e.g., large convolutional kernels, LayerScale), but also requires sufficient data support. In comparison, our model samples latent features from the posterior latent feature distribution, which increases the diversity of intra-class sample features and provides a more stable feature learning path under limited sample sizes. The EfficientNetV2 model balances depth, width, and resolution through compound scaling, and the model inference is more complex and requires fine tuning, whereas our model enhances generalization capability through latent feature distribution constraints.

In summary, the model proposed in this paper is based on the idea of variational inference. By enhancing the diversity of latent features, it makes the distribution of similar samples in the feature space more compact through the strategy of enhancing the diversity of latent features, and at the same time, the training strategy of incorporating prior knowledge enhances the inter-class discriminative ability of the model categorization in the feature space, thereby forming clear decision boundaries among different categories. Compared to current mainstream models, the proposed network architecture is more lightweight and also helps to improve the model’s ability to generalize in learning from small sample data.

### 3.3. Ablation Study

In this subsection, we conducted ablation experiments to investigate the effectiveness and robustness of each major network module in the proposed model in this paper for the image classification of *Dendrobium officinale*. We performed ablation experiments on three models, w/o Pos, w/o Pri, and the full-model (VIDE), to clearly demonstrate the impact of each component on the model. Specifically, w/o Pos does not enhance latent features through the posterior network, but only outputs predictions based on the prior network. Both w/o Pri and the full-model obtain posterior latent features through posterior networks by sampling them in regularized class latent feature probability distributions. The difference is that the posterior network in the full-model utilizes the prior knowledge from the w/o Pos model to enhance the posterior latent features. In contrast, w/o Pri sets values of the feature maps of all channels in the prior network to zero, and the prior knowledge is not transferred to the posterior network.

**Figure 5:**
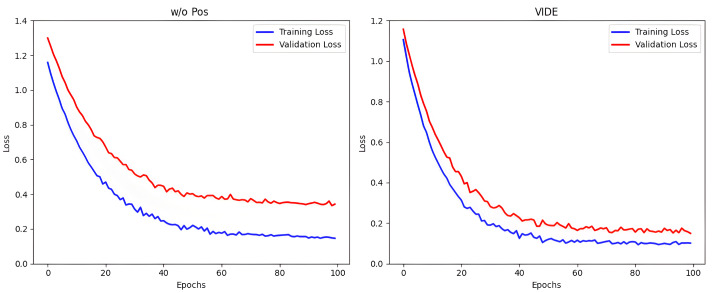
The loss function curves of w/o Pos and VIDE.

Table 6 presents the performance of w/o Pos, w/o Pri, and the full-model in the geographical origin identification task of *Dendrobium officinale*. From the results in the table, the full-model achieves higher accuracy compared to both w/o Pos and w/o Pri, confirming that the proposed model framework can effectively improve the performance of the algorithm. Specifically, both the full-model and the w/o Pri outperform the w/o Pos in terms of performance. This is because the posterior network, through the latent feature enhancement strategy, makes the distribution of the same class samples in the latent feature space more compact, mitigating the overfitting problems of the model with limited samples. This observation is further supported by the loss curves of the full-model and the w/o Pos shown in Figure 5. Furthermore, the full-model demonstrates superior performance over the w/o Pri model, which indicates that the prior knowledge can effectively enhance the posterior network’s sampling of the posterior latent features under the constraint of distributional regularization, enabling the model to achieve better performance in the task of classifying origins for *Dendrobium officinale*.

### 3.4. Analysis of the Impact of Sample Size on Model Performance

**Figure 6:**
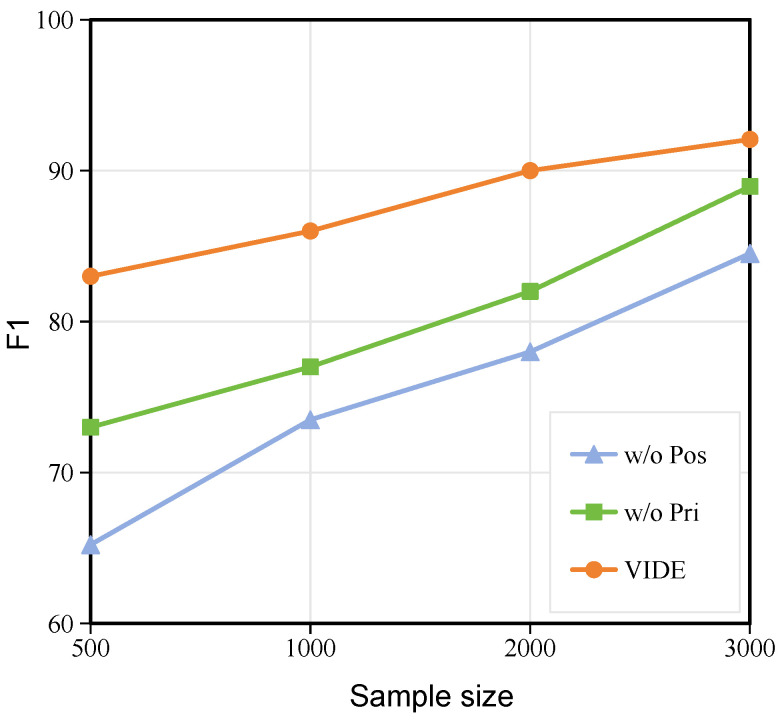
Effect of sample size on model performance.

In this subsection, we evaluate the performance of the VIDE model using training samples of varying sizes. As illustrated in Figure 6, we set the training sample sizes to 500, 1000, 2000, and 3000 to investigate the model’s performance under different data scales. The experimental results demonstrate that as the sample size increases, the classification accuracy of all three models shows an upward trend. However, the full-model (VIDE) consistently outperforms the ablated models, w/o Pos and w/o Pri, with its advantage being particularly pronounced at smaller sample sizes. Specifically, when the sample size is 500, the VIDE achieves an F1-score approximately 18% and 10% higher than w/o Pos and w/o Pri, respectively. Although this gap narrows when the sample size increases to 3000, the VIDE still maintains a performance lead of about 8% and 3%, respectively. This observation further validates the robustness of the VIDE model under data-scarce conditions.

On the one hand, the latent feature enhancement strategy of the posterior network effectively mitigates overfitting in small-sample scenarios, allowing both w/o Pri and the VIDE to outperform w/o Pos, which relies solely on the prior network. On the other hand, by incorporating prior knowledge to guide posterior feature sampling, the VIDE significantly improves the model’s discriminative capability for latent features, leading to more stable performance gains across different sample sizes. Furthermore, as the sample size increases, the performance improvement of all models gradually slows, indicating diminishing marginal returns when the data scale exceeds a certain threshold. Nevertheless, the VIDE continues to extract discriminative information from the data through its prior-posterior collaborative optimization mechanism.

In conclusion, both the ablation experiments and the sample size analysis demonstrate that the proposed VIDE model, by integrating prior knowledge with posterior feature enhancement, not only enhances the accuracy of *Dendrobium officinale* image classification but also improves the model’s adaptability across varying data scales.

### 3.5. Parameter Sensitivity Analysis

**Figure 7:**
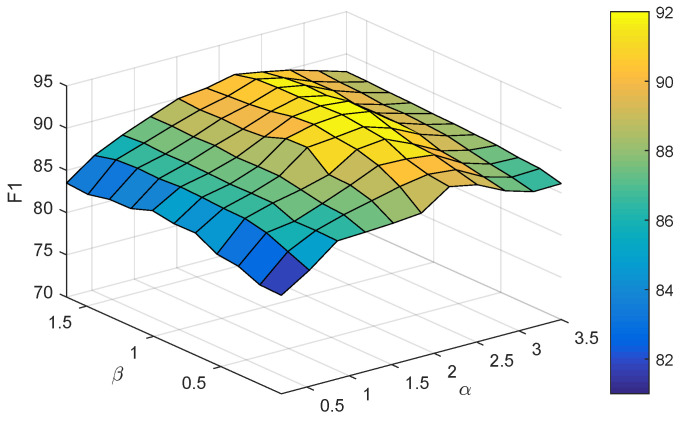
Impact of hyperparameters on model performance.

In this subsection, we conducted parameter sensitivity analysis experiments on the proposed model to further investigate the impact of hyperparameters α and β on the model performance. The experimental results of the parameter sensitivity analysis are shown in Figure 7. The hyperparameters α and β were searched in the grids $\left\{1.00,1.25,1.50,1.75,2.00,2.25,2.50,2.75,3.00,3.25,3.50\right\}$ and {0.05,0.30,0.45,0.60,0.75,0.90,1.05,1.20,1.35,1.50,1.65}, respectively.

The search range for the optimal value of hyperparameter α is $\left[2.0,2.5\right]$. Choosing an α value that is too high or too low negatively impacts model performance. The reason for this is that if α→∞, the optimization variables in the mean value network will tend to infinity. Generally, we want the weights of the network to obtain smaller values to enhance the generalization ability of the network, and use weight decay regularization method to achieve the above purpose. Therefore, if α is too large, it is contrary to the weight decay regularization method. However, if α→0, the latent feature regularization term in the loss function will cause the regions of the posterior latent feature distributions of the various types of samples to overlap, which adversely affects the sampling of posterior latent features and thus fails to motivate the model to generate clear decision boundaries.

The range for the optimal value of hyperparameter β is $\left[0.6,0.9\right]$. The model’s effectiveness diminishes if β is set beyond an optimal range. The reason for this is that if β→∞, it will make the posterior latent feature distributions of the various types of samples overlap, resulting in inter-class latent features interfering with each other, making it difficult to capture the intra-class latent features of the samples, and biasing the model’s learning focus. Conversely, if β→0, the gradient values of the relevant variables tend to infinity, which will cause the algorithm to fail to converge.

## 4. Discussion

Although conventional methods for identifying the geographical origin of *Dendrobium officinale*, such as expert morphological assessment and spectroscopic techniques, have played an important role in quality control, they face practical limitations. Traditional morphological approaches rely heavily on subjective expert judgment of traits such as stem morphology, texture, and color, which are susceptible to environmental variability and lack reproducibility. Spectroscopic techniques (e.g., NIRS, ATR-FTIR, NMR), though accurate, require costly instrumentation, destructive sampling, and operational expertise, constraining their use in field applications such as rural farms or market surveillance [34,35].

**Figure 8:**
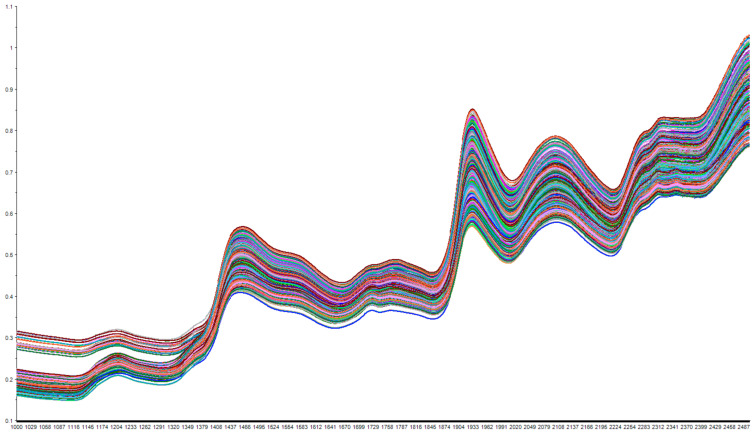
Near-infrared spectral analysis of *Dendrobium officinale* samples from different geographical origins. Each line represents a near-infrared spectrum of a powder sample. The vertical axis represents absorbance, and the horizontal axis represents wavelength (nm).

Figure 8 presents the near-infrared (NIR) spectra of *Dendrobium officinale* samples from different geographical origins. The dried stems of *Dendrobium officinale* were ground into powder and sieved to prepare uniform samples for NIR analysis. A total of 11 geographical origins were included, with 20 powder samples prepared from each origin, resulting in 220 powder samples in total. Measurements were conducted using a SupNIR-2750 spectrometer under controlled conditions (ambient temperature: 25 °C; humidity: 60%; spectral range: 1000–2500 nm; resolution: 1 nm; five repeated scans per sample). This process yielded a total of 1100 NIR spectra. The spectra show high similarity across samples from different regions, with consistent spectral profiles and absorption trends, which aligns with findings in studies focusing on chemical component prediction or species discrimination using NIRS [9,20,36]. NIR spectroscopy captures overtone and combination vibrations of hydrogen-containing groups (e.g., C–H, O–H), which are abundant in polysaccharides, alkaloids, and amino acids—key constituents of *Dendrobium officinale*. Prominent absorption peaks are observed around 1380–1520 nm (associated with C–H combinations), 1880–1960 nm, and 2020–2150 nm. The consistent spectral shapes suggest underlying chemical similarities among samples from different regions, making it difficult to differentiate origins based solely on peak intensity, shape, or position, a challenge also noted in origin identification studies [37].

The subtle spectral variations observed likely arise from complex interactions between geographical, climatic, and cultivation factors. Although NIR spectroscopy combined with advanced chemometrics (e.g., PLS-DA, SVM, CARS-PLS) has demonstrated efficacy in predicting specific bioactive compounds (e.g., antioxidant properties [9,36], saponins, mannitol [20]) or in comprehensive quality evaluation and origin differentiation [34,37], effective discrimination for precise geographical origin traceability often still requires sophisticated modeling to extract meaningful features from complex spectral data [37,38]. This underscores both the challenges and opportunities in using chemical profiles for origin traceability. Recent advances have explored multi-spectroscopy fusion [34] and deep learning models like ResNet applied to spectral data [38] or 2D correlation spectroscopy images [39] to improve discrimination performance for *Dendrobium* species or origins. While these methods show promise, they typically remain reliant on spectral instrumentation.

In contrast, the proposed VIDE framework leverages the non-destructive, low-cost, and accessible nature of image-based methods, combined with the discriminative power of variational inference and deep learning. Unlike spectroscopy, which relies on predefined chemical features or requires specialized equipment and sample preparation [9,20,35,36,37], our model learns discriminative visual phenotypic patterns directly from images captured using conventional smartphones, making it highly scalable and practical for real-world use, particularly in resource-limited settings. Furthermore, the VIDE architecture is inherently flexible, enabling the incorporation of additional phenotypic traits, such as fine texture, geometric shape or spatial structure, without the need for structural modification. This allows for the continuous enhancement of the model as new data becomes available.

By bridging computer vision and botanical phenotyping, the VIDE model not only offers superior accuracy and efficiency but also provides an adaptable platform for integrating multimodal data in the future. This approach holds significant promise for non-destructive, high-throughput origin identification and quality assurance of *Dendrobium officinale*. As demand for this medicinal and edible plant continues to grow, we plan to expand our dataset to include more regions and cultivation conditions, further improving the model’s reliability and generalizability to meet industry needs.

## 5. Conclusions

This study introduces a Variational Inference-enabled Data-Efficient Learning (VIDE) framework to address the challenge of limited sample sizes in the geographical authentication of *Dendrobium officinale*. The model employs a dual-network probabilistic architecture in which posterior network sampling, guided by prior knowledge transfer, produces regularized latent features that reduce intra-class dispersion and inter-class confusion. By combining a cost-effective mobile imaging dataset with a lightweight network design, the system enables non-destructive origin identification without requiring specialized instrumentation. Empirical results demonstrate that VIDE outperforms benchmark models in F1-score and accuracy, confirming its enhanced capability for discriminative feature extraction.

More importantly, the VIDE model provides the *Dendrobium officinale* industry with a low-cost, readily deployable tool for geographical origin certification that can be seamlessly integrated into production and distribution chains. This technology helps address widespread market issues such as misrepresentation of inferior products as high-quality ones and product adulteration, thereby improving quality assurance and supporting brand integrity. It carries significant practical implications for promoting standardization and sustainable development within the *Dendrobium officinale* industry.

## Figures and Tables

**Table 1:** Origin information of the *Dendrobium officinale* samples.

Batch ID	Number of Samples	Planting Region	Cultivation Method	Soil Type	Altitude (m)
a	200	Libo, Guizhou	Wild-simulated	Red Earth	300–400
b	200	Libo, Guizhou	Greenhouse	Red Earth	300–400
c	200	Baoshan, Yunnan	Wild-simulated	Red Earth	1000–1100
d	200	Baoshan, Yunnan	Greenhouse	Red Earth	1000–1100
e	200	Yueqing, Zhejiang	Wild-simulated	Red Earth	300–400
f	200	Yueqing, Zhejiang	Greenhouse	Red Earth	300–400
g	200	Shaoguan, Guangdong	Wild-simulated	Red Earth	300–400
h	200	Shaoguan, Guangdong	Greenhouse	Red Earth	300–400
i	200	Laibin, Guangxi	Wild-simulated	Red Earth	300–400
j	200	Laibin, Guangxi	Greenhouse	Red Earth	300–400
k	200	Yingtan, Jiangxi	Wild-simulated	Red Earth	300–400

**Table 2:** Physical characteristics of the *Dendrobium officinale* samples.

Batch ID	Length (cm)	Diameter (cm)	Weight (g)	Internode Length (cm)	Surface Color
a	17.11 ± 2.22	0.16 ± 0.03	0.56 ± 0.19	1.35 ± 0.23	Yellowish-green
b	16.06 ± 2.91	0.20 ± 0.03	0.52 ± 0.16	2.06 ± 0.41	Yellowish-green
c	14.28 ± 2.08	0.20 ± 0.03	0.67 ± 0.18	1.30 ± 0.21	Yellowish-green
d	8.66 ± 0.87	0.24 ± 0.03	0.64 ± 0.18	1.08 ± 0.19	Yellowish-green
e	12.98 ± 2.13	0.15 ± 0.02	0.29 ± 0.10	1.65 ± 0.28	Yellowish-green
f	23.55 ± 3.66	0.27 ± 0.03	2.53 ± 0.73	1.55 ± 0.27	Yellowish-green
g	23.64 ± 3.54	0.18 ± 0.03	0.75 ± 0.20	1.79 ± 0.30	Yellowish-green
h	21.49 ± 1.89	0.25 ± 0.03	1.67 ± 0.36	1.37 ± 0.22	Yellowish-green
i	16.37 ± 2.08	0.16 ± 0.03	0.43 ± 0.15	1.93 ± 0.36	Yellowish-green
j	18.40 ± 3.38	0.23 ± 0.03	1.34 ± 0.34	1.57 ± 0.26	Yellowish-green
k	11.42 ± 1.90	0.25 ± 0.02	0.82 ± 0.23	1.23 ± 0.20	Yellowish-green
ANOVA *p*-value	<0.001	<0.001	<0.001	<0.001	

**Table 3:** Performance results on the greenhouse cultivation dataset.

Method	Precision (%)	Recall (%)	F1 (%)	Accuracy (%)
Alexnet	77.24	76.62	76.93	77.21
VGGNet	80.61	80.53	80.57	80.93
GoogleNet	82.17	82.66	82.41	83.14
DenseNet-121	86.09	85.78	85.93	86.75
ResNet-18	84.33	83.45	83.89	84.69
ResNet-50	87.65	86.81	87.23	88.02
ConvNeXt	91.80	91.54	91.67	92.38
EfficientNetV2	92.28	91.93	92.10	92.86
Vision Transformer (ViT)	89.52	88.37	88.94	90.11
VIDE (Ours)	95.41	95.83	95.62	96.53

**Table 4:** Performance results on the wild-simulated cultivation dataset.

Method	Precision (%)	Recall (%)	F1 (%)	Accuracy (%)
Alexnet	75.27	73.65	74.45	74.92
VGGNet	78.43	77.88	78.15	78.85
GoogleNet	81.76	81.12	81.44	82.07
DenseNet-121	85.88	85.36	85.62	86.73
ResNet-18	84.15	82.27	83.20	84.14
ResNet-50	87.39	86.44	86.91	87.79
ConvNeXt	90.66	90.61	90.63	91.68
EfficientNetV2	91.54	90.97	91.25	92.36
Vision Transformer (ViT)	88.67	87.26	87.96	88.79
VIDE (Ours)	94.37	93.92	94.14	95.21

**Table 5:** Performance results on the mixed dataset with greenhouse and wild-simulated samples.

Method	Precision (%)	Recall (%)	F1 (%)	Accuracy (%)
Alexnet	73.38	71.94	72.65	73.82
VGGNet	77.16	75.33	76.23	77.19
GoogleNet	79.65	78.48	79.06	79.87
DenseNet-121	83.34	81.12	82.22	83.04
ResNet-18	81.52	79.29	80.39	81.25
ResNet-50	84.71	82.74	83.71	84.66
ConvNeXt	86.03	87.55	86.78	87.58
EfficientNetV2	88.47	87.63	88.05	88.91
Vision Transformer (ViT)	86.29	84.75	85.51	86.64
VIDE (Ours)	91.51	92.63	92.07	92.93

**Table 6:** Ablation analysis of VIDE model components.

Method	Precision (%)	Recall (%)	F1-Score (%)
w/o Pos	85.77	83.29	84.51
w/o Pri	89.34	88.56	88.95
VIDE (Ours)	91.51	92.63	92.07

## Data Availability

The data that support the findings of this study are available upon a reasonable request.
